# Buckling of Carbon Nanotubes: A State of the Art Review

**DOI:** 10.3390/ma5010047

**Published:** 2011-12-28

**Authors:** Hiroyuki Shima

**Affiliations:** Division of Applied Physics, Faculty of Engineering, Hokkaido University, Kita-13, Nishi-8, Kita-ku, Sapporo, Hokkaido 060-8628, Japan; E-Mail: shima@eng.hokudai.ac.jp; Tel.: +81-11-706-6624; Fax: +81-11-706-6859

**Keywords:** nanocarbon material, nanomechanics, nonlinear deformation

## Abstract

The nonlinear mechanical response of carbon nanotubes, referred to as their “buckling" behavior, is a major topic in the nanotube research community. Buckling means a deformation process in which a large strain beyond a threshold causes an abrupt change in the strain energy *vs.* deformation profile. Thus far, much effort has been devoted to analysis of the buckling of nanotubes under various loading conditions: compression, bending, torsion, and their certain combinations. Such extensive studies have been motivated by (i) the structural resilience of nanotubes against buckling and (ii) the substantial influence of buckling on their physical properties. In this contribution, I review the dramatic progress in nanotube buckling research during the past few years.

## 1. Introduction: Appeal of Nanocarbon Materials

Carbon is a rare substance that takes highly diverse morphology. When carbon atoms form a three-dimensional structure, their glittering beauty as diamonds is captivating. When aligned in a two-dimensional plane, they make up just black graphite and lose their sparkle. In addition to these “macro"-scopic carbon materials, several “nano"-carbon materials have been discovered in the past few decades, opening up new horizons in material sciences. It all began with the C${}_{60}$ molecule (fullerene), whose existence was predicted by Osawa [1] in 1970 and was discovered by Kroto *et al*. [2] in 1985. Subsequent studies, including those on carbon nanotubes by Iijima [3] in 1991 [4] and on graphene by Novoselov *et al*. [5] in 2004, have had a tremendous impact and driven developments in science and engineering around the turn of the century [6,7,8,9].

Among the three types of nanocarbon materials, carbon nanotubes are attracting the greatest attention in both industry and academia. Research on carbon nanotubes has brought out two characteristics not usually seen in other fields. First and foremost is the sheer breadth of the research, which encompasses physics, chemistry, materials science, electronics, and life science. The second characteristic is that basic research and applied research are extremely close to each other. A succession of phenomena of interest to scientists has been discovered like a treasure chest, each leading to an innovative application or development. Nowadays, it is difficult even for professionals in the nanotube research community to understand the progress being made outside of their field of expertise.

One of the reasons why carbon nanotubes offer huge potential is the fact that mechanical deformation causes considerable changes in electronic, optical, magnetic, and chemical properties. Thus, many studies on new technologies to utilize the correlation between deformation and properties are underway in various fields. For example, studies of nanoscale devices based on the change in electrical conductivity or optical response resulting from deformation are one of the most popular trends in nanotechnology. Another important reason for nanotube research diversity is the concomitance of structural resilience and small weight, making realizable ultrahigh-strength materials for utilization in super-high-rise buildings and large aerospace equipment. Furthermore, applications of these low-density substances for aircraft and automobile parts will raise fuel efficiency and save energy, as well as dramatically reduce exhaust gas emissions and environmental impact.

With this background in mind, I shall review recent development in a selected area of nonlinear mechanical deformation, the “buckling" of carbon nanotubes [10,11]. Section 2 provides a concise explanation of the terminology of buckling, followed by a survey of different approaches used in nanotube buckling investigations. Section 3 details the two most interesting features observed during nanotube buckling process, *i.e.*, the structural resilience and sensitivity of nanotube properties against buckling. Section 4 to Section 8 are the main part of this paper, illustrating nanotube buckling under axial compression (Section 4), radial compression (Section 5), bending (Section 6, Section 7), and torsion (Section 8). Section 9 presents a universal scaling law that describes different buckling modes of nanotubes in a unified manner. The article is closed by Section 10 that describes several challenging problems whose solutions may trigger innovation in the nanotube research community. The list of references (over 210 inclusing Notes) is fairly extensive, although by no means all inclusive. To avoid overlap with the existing excellent reviews [12,13,14], results reported within the past few years are featured in words that nonspecialists can readily understand.

## 2. Background of Nanotube Buckling Research

**Figure 1:**
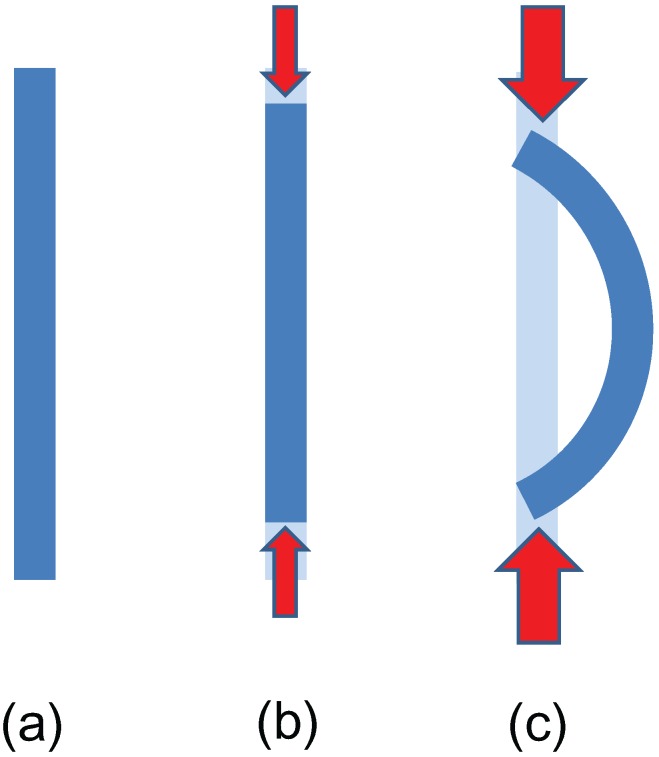
Schematic diagram of buckling of an elastic beam under axial compression: (**a**) pristine beam; (**b**) axial compression for a small load; (**c**) buckling observed beyond a critical load.

The term “buckling” means a deformation process in which a structure subjected to high stress undergoes a sudden change in morphology at a critical load [15]. A typical example of buckling may be observed when pressing opposite edges of a long, thin elastic beam toward one another; see Figure 1. For small loads, the beam is compressed in the axial direction while keeping its linear shape [Figure 1(b)], and the strain energy is proportional to the square of the axial displacement. Beyond a certain critical load, however, it suddenly bends archwise [Figure 1(c)] and the relation between the strain energy and displacements deviates significantly from the square law. Besides axial compression, bending and torsion give rise to buckling behaviors of elastic beams, where the buckled patterns strongly depend on geometric and material parameters. More interesting is the elastic buckling of structural pipe-in-pipe cross sections under hydrostatic pressure [16,17]; pipe-in-pipe (*i.e.*, a pipe inserted inside another pipe) applications are promising for offshore oil and gas production systems in civil engineering.

The above argument on macroscopic elastic objects encourages to explore what buckled patterns are obtained in carbon nanotubes. Owing to their nanometric scales, similarities and differences in buckled patterns compared with macroscopic counterparts should not be trivial at all. This complexity has motivated tremendous efforts toward the buckling analysis of carbon nanotubes under diverse loading conditions: axial compression [18,19,20,21,22,23,24,25,26,27,28], radial compression [29,30,31,32,33,34,35,36,37,38,39,40,41,42,43,44,45,46,47,48,49,50,51,52,53,54,55,56,57,58,59,60,61,62,63], bending [41,64,65,66,67,68,69,70,71], torsion [72,73,74,75,76,77], and their certain combinations [78,79,80,81,82]. Such extensive studies have been driven primarily by the following two facts. One is the excellent geometric reversibility of nanotubes against mechanical deformation; that is, their cylindrical shapes are reversible upon unloading without permanent damage to the atomic structure. In addition, carbon nanotubes exhibit high fatigue resistance; therefore, they are the promising medium for the mechanical energy storage with extremely high energy density [83]. The other fact is the substantial influence of buckling on their physical properties. It was recently shown that, just as one example, carbon nanotubes undergoing an axial buckling instability have potential utility as a single-electron transistor [84,85,86] and can play a crucial role in developing nanoelectromechanical systems.

**Figure 2:**
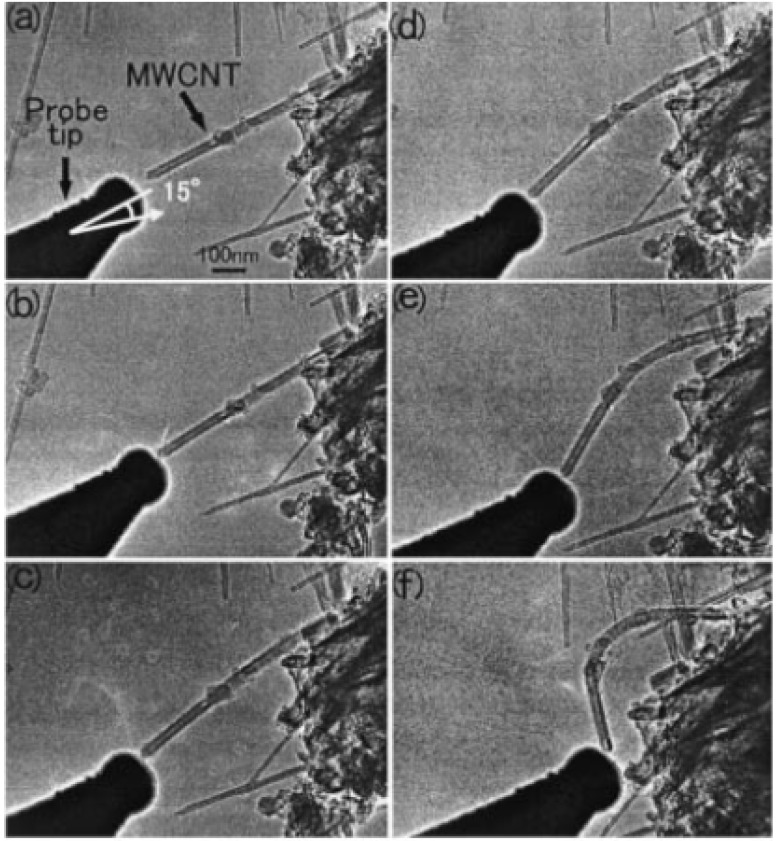

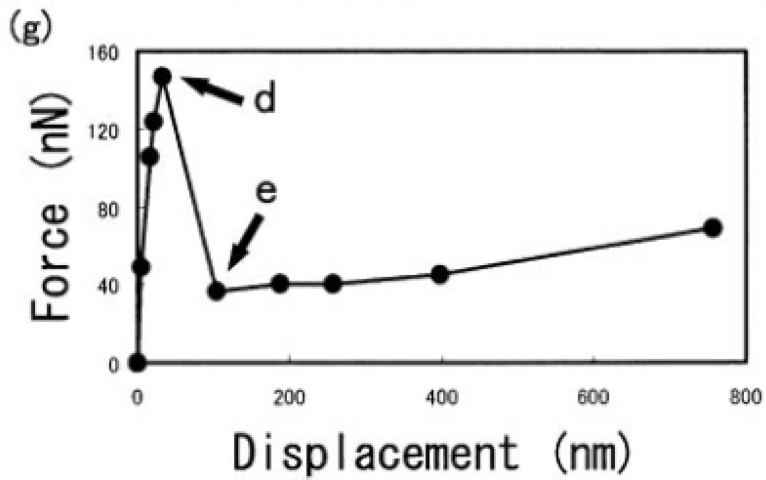
(**a**)–(**f**) Series of TEM images of deformation processes for MWNTs initiated by applying compressive force in the sample direction; (**g**) Force–displacement diagram. The points indicated by arrows correspond to the TEM images in (**d**) and (**e**). Reprinted from Reference [88].

Microscopy measurements are powerful means of examining the nonlinear response of nanotubes against external loading. For instance, atomic force microscopy (AFM) was utilized to reveal the force–distance curve of nanotubes while buckling [87]. More direct characterizations of nanotube buckling were obtained by *in situ* transmission electron microscopy (TEM) [88,89,90], as partly demonstrated in Figure 2 (see Section 4.2). However, the experimental investigation of nanotube buckling remains a challenge because of difficulties in manipulation at the nanometric scale. This is a reason why both theoretical and numerical approaches have played an important role in exploring the buckling behavior of nanotubes. In theoretical studies, carbon nanotubes are commonly treated as beams or thin-shell tubes with certain wall thickness and elastic constants [36,44,91,92,93,94,95,96,97,98,99,100]. Such continuum approximations are less computationally expensive than atomistic approaches; moreover, the obtained formulations can be relatively simple in general. It is noteworthy that, by substituting appropriate values into elastic constants, continuum-mechanics approaches provide a unified framework [98] that accounts for the critical buckling strains and buckled morphologies under various loading conditions covering compression, bending, torsion, *etc*.

## 3. Resilience and Sensitivity to Buckling

Special emphasis should be placed on the fact that carbon nanotubes exhibit many intriguing postbuckling morphologies: Radial corrugations (see Section 5.2) and surface rippling (Section 9) are typical examples. One of the most outstanding features of postbuckled nanotubes is their geometric reversibility upon unloading. Indeed, experiments have shown that the buckling deformation can be completely recovered when the load is removed [64,65,66,101,102,103,104]. The marked structural resilience is primary because of (i) the large in-plane rigidity of graphene sheets rather than low bending rigidity [105] and (ii) the intrinsic hollow geometry with extremely large aspect ratio that carbon nanotubes exhibit. It was suggested that the resilience makes it possible to use the nanotubes as a gas [76,106,107] or water pipeline [108] whose permeability can be tuned by mechanical deformation.

Apart from the structural resilience, the sensitivity of carbon nanotube properties to buckling is worthy of attention. In fact, the breakdown of the structural symmetry resulting from the buckling triggers sudden changes in physical and mechanical properties of nanotubes, including thermal conductivity reduction [109,110,111], a radial breathing-mode frequency shift [112], the emergence of interwall sp${}^{3}$ bondings [113], and electromechanical responses under bending [114,115,116] and torsion [117,118], to name a few. In addition, the buckling-induced reduction in nanotube stiffness not only impairs the ability of nanotubes to sustain external loadings as reinforced fibers in nanocomposites [102,103] but also gives rise to large uncertainties in the vibration behavior of nanotubes as nanoscale resonators [66,119,120]. These buckling-property relations can significantly influence the performance of nanotubes as structural or functional elements, thus implying the need of a huge amount of effort that has been made for the study of nanotube buckling.

## 4. Axial Compression Buckling

### 4.1. Shell Buckling or Column Buckling?

Buckled patterns of single-walled nanotubes (SWNTs) under axial compression depend on their aspect ratio [18,21,36], which equals the ratio of length to diameter of nanotubes. Roughly, a thick and short SWNT (*i.e.*, with small aspect ratio) undergoes shell buckling while keeping a straight cylindrical axis, whereas a thin and long one tends to exhibit a series of shell and column (or Euler) buckling.

**Figure 3:**
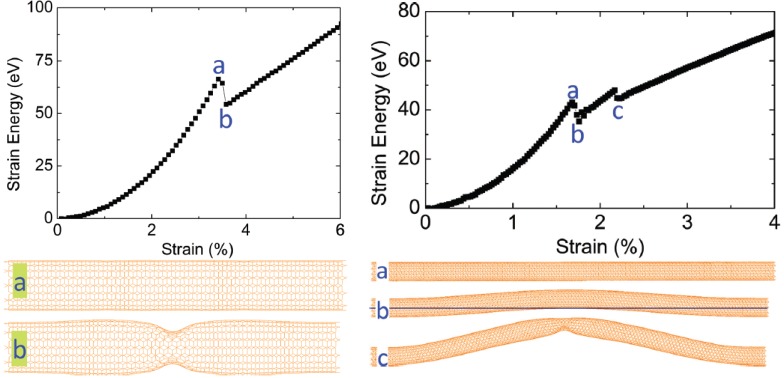
Axially buckled SWNT pattern deduced from molecular dynamics simulations. [Left] Upper panel: Energy-strain curve of a (10,10) SWNT with a length of 9.6 nm under axial compression. Lower panel: Typical tube geometry (**a**) before and (**b**) after buckling, respectively. [Right] Upper panel: Curve of a compressed (10,10) SWNT with 29.5 nm length. Lower panel: Snapshots of the tube (**a**) before buckling; (**b**) after column buckling; and (**c**) undergoing shell buckling. Reprinted from Reference [121].

The shell buckling process is depicted in the left panel of Figure 3 [121], where a (10,10) SWNT with a length of 9.6 nm and an aspect ratio of ∼7 was chosen [122,123]. It is seen that the strain energy increases quadratically with strain at the early prebuckling stage. At a critical strain of 3.5%, a sudden drop in energy is observed [124,125], corresponding to the occurrence of shell buckling. During the postbuckling stage, the strain energy exhibits a linear relationship with strain. The linear growth in energy is understood by the primary role of the change in carbon-carbon (C-C) bond angles, rather than that of the bond length variation, in determining the energy-strain relation after buckling. Detailed analyses in Reference [121] showed that within the post-buckling regime, the variation in bond angles between neighboring C-C bonds becomes significant while C-C bond lengths show less variation; this results in an almost constant axial stress, as deduced from Figure 3.

With increasing aspect ratio, the buckling mode switches to a column buckling mode owing to the increased flexibility of the tube. Column buckling is seen in the right panel of Figure 3 for a much longer (10,10) SWNT (29.5 nm in length with an aspect ratio of ∼22). A sudden drop in strain energy occurs at a strain level of 1.6%, beyond which the center is displaced in a transverse direction away from its original cylindrical axis. When the already column-buckled SWNT is further compressed, the structure curls further and a second drop in strain energy is observed at a strain level of 2.2%. The second drop corresponds to the onset of another buckling, which is responsible for releasing the excess strain energy. The tube geometry at this point indicates that the SWNT undergoes shell buckling. With further axial compression, a linear relationship is observed between energy and strain. This result indicates a column- to shell-buckling transition of SWNTs with large aspect ratio [68,126].

It is important to note that the initial buckling modes, corresponding to the first drop in energy-strain curve, are different between large- and small-aspect-ratio SWNTs. This fact necessitates an examination of the validity of continuum-mechanics models for the buckling of SWNTs. Careful assessments of the continuum approximations have been reported [98,99,100,125,127], indicating the need to properly use different models depending on the aspect ratio. As to their consistency with atomistic simulation results, readers can refer to Reference [125] in which a list of critical strain data under different conditions is detailed.

### 4.2. Force–Displacement Curve

We now turn to experimental facts [128]. Because of the difficulty in sample preparation and manipulation, only a few attempts have been made to perform axial buckling measurements [88,89,90]. In particular, experimental realization of shell buckling under compression has largely behind, though its signature has been obtained via nanoindentation [129]. Hence in the following discussion, we focus our attention to the column buckling measurements.

The pioneering work [88] is presented in Figure 2; The TEM images of (a)–(f) clarify a series of deformation processes for multiwalled nanotubes (MWNTs) initiated by applying a compressive force in the nearly axial direction [130]. Figure 2(g) shows the corresponding force–displacement diagram. The force at the initial stage is almost proportional to the displacement [left to the point (d)], indicating the elastic region, followed by an abrupt decrease at (e). The two points indicated in Figure 2(g) correspond to the TEM images in panels (d) and (e), respectively. In Figure 2(g), the curve right to the point (e) maintains a slightly upward slope. The reason for this post-buckling strength may be due to sequential emergence of different buckling patterns with increasing the displacement.

**Figure 4:**
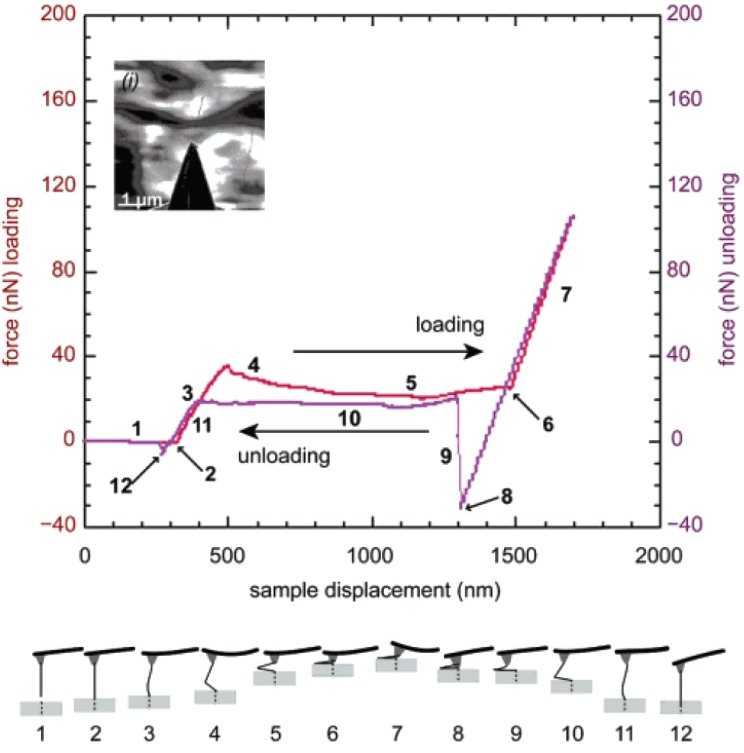
[Top] Force-displacement curve of an MWNT with an aspect ratio of ∼80 under cyclic axial loading. This inset shows a microscopy image; [Bottom] Schematic of the change in the MWNT configuration during the buckling process. The labels correspond to those indicated in the force-displacement curve. Reprinted from Reference [87].

A more sweeping measurement on the nanotube resilience was performed for the MWNT with a higher aspect ratio (∼80). Figure 4 shows the resulting force–displacement curve and graphical illustration of the buckling process [87]. An important observation is a negative stiffness region (labeled by “4" in the plot) that begins abruptly. The sharp drop in force with increasing axial strain, observed at the boundary of regions (3) and (4), is attributed to the kinking of the MWNT as depicted in the lower panel. After the kinking takes place, the system is mechanically instable; this behavior is consistent with the mechanics of kinking described in References [18,88]. The instability seen in region (4) is reproducible through cyclic compression, which opens up the possibility of harnessing the resilient mechanical properties of MWNTs for novel composites [131].

## 5. Radial Compression Buckling

### 5.1. Uniaxial Collapse of SWNTs

Radial pressure can yield a distinct class of buckling, reflecting the high flexibility of graphene sheets in the normal direction. In fact, radial stiffness of an isolated carbon nanotube is much less than axial stiffness [132], which results in an elastic deformation of the cross section on applying hydrostatic pressure [29,30,31,32,33,34,37,38,39,40,42,43,45,47] or indentation [59,62,133]. Experimental and theoretical studies, focused on SWNTs and their bundles, revealed flattening and polygonalization in their cross section under pressures on the order of a few gigapascals [29,32]. Nevertheless, existing results are rather scattered, and we are far away from a unified understanding; for example, the radial stiffness of nanotubes estimated thus far vary by up to three orders of magnitude [34,37,38,43,46,59,62,132].

**Figure 5:**
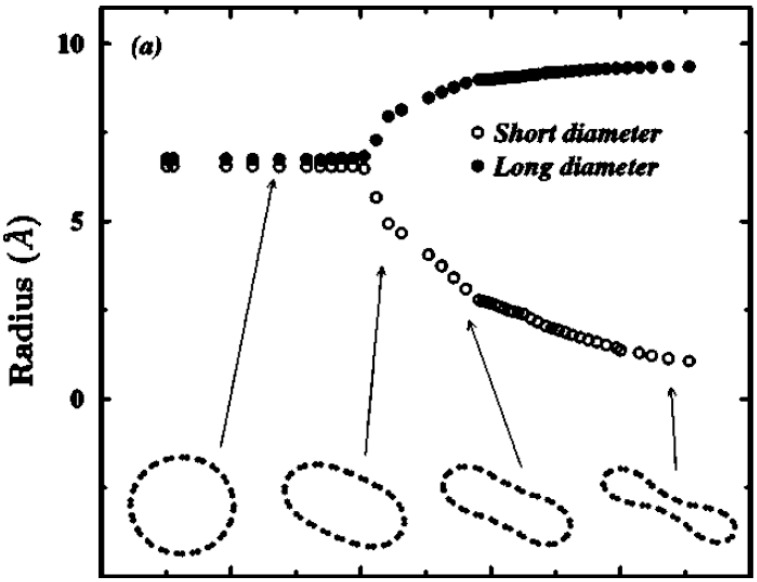
Long and short diameters of a (10,10) SWNT as a function of applied hydrostatic pressure. The shape of the cross section at some selected pressures is plotted at the bottom of the figure. Reprinted from Reference [38].

The overall scenario of SWNT deformation under hydrostatic pressure is summarized in Figure 5 [38]. With increasing pressure, cross sections of SWNTs deform continuously from circular to elliptical, and finally to peanut-like configurations [38,42,134,135]. The radial deformation of carbon nanotubes strongly affects their physical and structural properties. For instance, it may cause semiconductor-metal transition [136,137], optical response change [138], and magnetic moment quenching [139] in nanotubes. From a structural perspective, the radial collapse can give rise to interwall sp${}^{3}$ bonding between adjacent concentric walls [140,141], which may increase nanotube stiffness and therefore be effective for high-strength reinforced composites [142,143,144,145].

**Figure 6:**
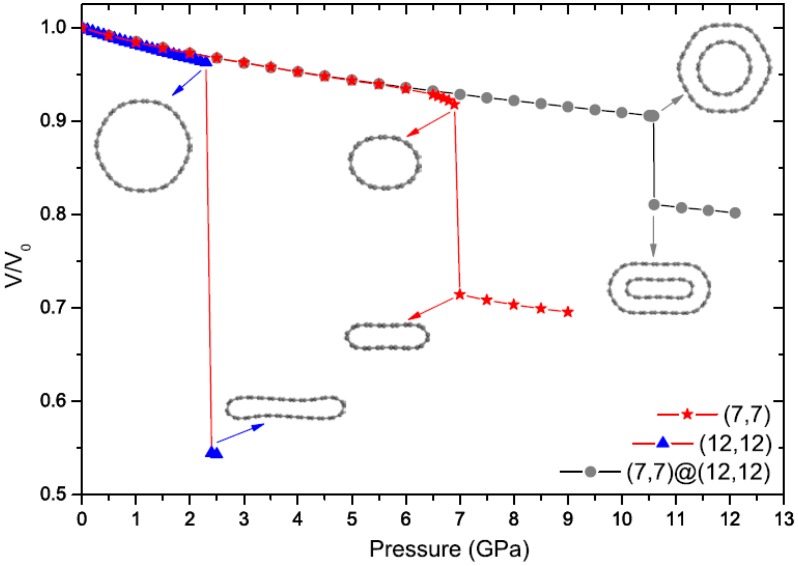
Change in the relative volume of the (7,7)@(12,12) DWNT bundle and the corresponding SWNT bundles as a function of hydrostatic pressure. Reprinted from Reference [146].

A bundle of nanotubes (*i.e.*, an ensemble of many nanotubes arranged parallel to each other) can exhibit similar radial collapse patterns to those of an isolated nanotube under hydrostatic pressure. Figure 6 shows [146] the volume change of a bundle of (7,7) SWNTs and a bundle of (12,12) SWNTs as a function of the applied hydrostatic pressure; the data for a bundle of (7,7)@(12,12) double-walled nanotubes (DWNTs) is also shown in the same plot. The (12,12) SWNT bundle, for instance, collapses spontaneously at a critical pressure of 2.4 GPa, across which the cross section transforms into a peanut-like shape. Two other bundles provide higher critical pressures, as follows from the plot. An interesting observation is that the transition pressure of the (7,7) tube, which is nearly 7.0 GPa when the tube is isolated, becomes higher than 10.5 GPa when it is surrounded by the (12,12) tube. This means that the outer tube acts as a “protection shield" and the inner tube supports the outer one and increases its structural stability; this interpretation is consistent with the prior optical spectroscopic measurement [147]. This effect, however, is weakened as the tube radius increases owing to the decreasing radial stiffness of SWNTs.

### 5.2. Radial Corrugation of MWNTs

In contrast to the intensive studies on SWNTs (and DWNTs), radial deformation of MWNTs remains relatively unexplored. Intuitively, the multilayered structure of MWNTs is expected to enhance the radial stiffness relative to a single-walled counterpart. However, when the number of concentric walls is much greater than unity, outside walls have large diameters, so external pressure may lead to a mechanical instability in the outer walls. This local instability triggers a novel cross-sectional deformation, called radial corrugation [53], of MWNTs under hydrostatic pressure.

**Figure 7:**
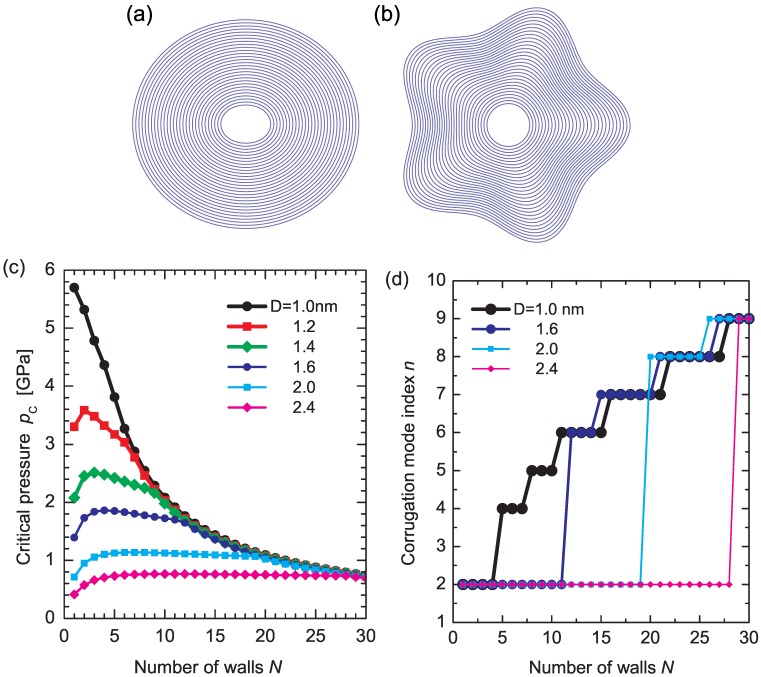
(**a**) Cross-sectional views of (**a**) elliptic (n=2); and (**b**) corrugated (n=5) deformation modes; The mode index *n* indicates the wave number of the deformation mode along the circumference; (**c**) Wall-number dependence of critical pressure $p_{c}$. Immediately above $p_{c}$, the original circular cross section of MWNTs gets radially corrugated; (**d**) Stepwise increase in the corrugation mode index *n*. Reprinted from References [53,61].

Figure 7(a,b) illustrates MWNT cross-sectional views of two typical deformation modes: (a) elliptic (n=2); and (b) corrugation (n=5) modes. In the elliptic mode, all constituent walls are radially deformed. In contrast, in the corrugation mode, outside walls exhibit significant deformation, whereas the innermost wall maintains its circular shape. Which mode will be obtained under pressure depends on the number of walls, N, and the core tube diameter *D* of the MWNT considered. In principle, larger *N* and smaller *D* favor a corrugation mode with larger *n*.

Figure 7(c) shows the critical buckling pressure $p_{c}$ as a function of *N* for various values of *D*. An initial increase in $p_{c}$ at small *N* (except for *D* = 1.0 nm) is attributed to the enhancement of radial stiffness of the entire MWNT by encapsulation. This stiffening effect disappears with further increase in *N*, resulting in decay or convergence of $p_{c}\left(N\right)$. A decay in $p_{c}$ implies that a relatively low pressure becomes sufficient to produce radial deformation, thus indicating an effective “softening" of the MWNT. The two contrasting types of behavior, stiffening and softening, are different manifestations of the encapsulation effect of MWNTs. It is noteworthy that practically synthesized MWNTs often show *D* larger than those presented in Figure 7(c). Hence, $p_{c}\left(N\right)$ of an actual MWNT lies at several hundreds of megapascals, as estimated from Figure 7(c). Such a degree of pressure applied to MWNTs is easily accessible in high-pressure experiments [60,148,149], supporting the feasibility of our theoretical predictions.

Figure 7(d) shows the stepwise increases in the corrugation mode index *n*. For all *D*, the deformation mode observed just above $p_{c}$ abruptly increases from n=2 to n≥4 at a certain value of *N*, followed by the successive emergence of higher corrugation modes with larger *n*. These successive transitions in *n* at N≫1 originate from the mismatch in the radial stiffness of the innermost and outermost walls. A large discrepancy in the radial stiffness of the inner and outer walls results in a maldistribution of the deformation amplitudes of concentric walls interacting via vdW forces, which consequently produces an abrupt change in the observed deformation mode at a certain value of *N*.

**Figure 8:**
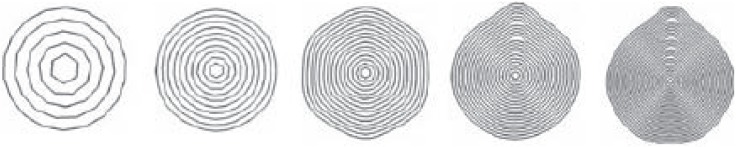
Cross-sectional views of relaxed MWNTs indexed by (2,8)/(4,16)/*…*/(2*n*,8*n*). The wall numbers *n* are 5, 10, 15, 20, and 25 from left to right, and all the MWNTs are 20 nm long. Reprinted from Reference [63].

Other types of radial deformation arise when deviate the interwall spacings of MWNTs from the vdW equilibrium distance (∼0.34 nm) [63,150,151,152]. The simulations show that the cross sections stabilized at polygonal or water-drop-like shapes, depending on the artificially expanded interwall spacings [153]. Figure 8 depicts the cross-sectional configurations of relaxed MWNTs. It is seen that the 15-walled tube is stabilized at a polygonal cross-sectional configuration with six rounded corners. For the 20- and 25-walled ones, the configuration becomes asymmetric, featuring a water-drop-like morphology.

From an engineering perspective, the tunability of the cross-sectional geometry may be useful in developing nanotube-based nanofluidic [154,155,156] or nanoelectrochemical devices [157,158] because both utilize the hollow cavity within the innermost tube. Another interesting implication is a pressure-driven change in the quantum transport of *π* electrons moving along the radially deformed nanotube. It has been known that mobile electrons whose motion is confined to a two-dimensional, curved thin layer behave differently from those on a conventional flat plane because of an effective electromagnetic field [159,160,161,162,163,164] that can affect low-energy excitations of the electrons. Associated variations in the electron-phonon coupling [165] and phononic transport [166] through the deformed nano-carbon materials are also interesting and relevant to the physics of radially corrugated MWNTs.

## 6. Bend Buckling of SWNTs

### 6.1. Kink Formation

**Figure 9:**
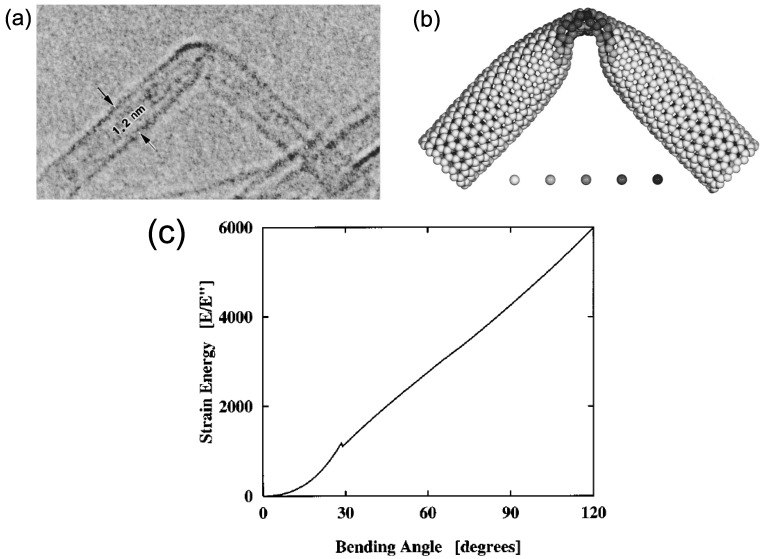
(**a**) Kink structure formed in an SWNT with diameters of 1.2 nm under bending. The gap between the tip of the kink and the upper wall is about 0.4 nm; (**b**) Atomic structure around the kink reproduced by computer simulations. The shaded circles beneath the tube image express the local strain energy at the various atoms, measured relative to a relaxed atom in an infinite graphene sheet. The strain energy scale ranges from 0 to 1.2 eV/atom, from left to right; (**c**) Total strain energy (in dimensionless units) of an SWNT of diameter ∼1.2 nm as a function of the bending angle up to 120${}^{\circ }$. The dip at ∼30${}^{\circ }$ in the curve is associated with the kink formation. Reprinted from Reference [64].

The buckling of SWNTs under bending was pioneered in 1996 [64] using high-resolution electron microscopes and molecular-dynamics (MD) simulation. Figure 9(a) shows a TEM image of a bent SWNT with a diameter of 1.2 nm [64]. By bending an initially straight SWNT, its outer and inner sides undergo stretching and compression, respectively. As a result, it develops a single kink in the middle, through which the strain energy on the compressed sides is released. Upon removal of the bending moment, it returns to the initial cylindrical form completely without bond breaking or defects. This observation clearly proves that SWNTs possess extraordinary structural elastic flexibility.

Figure 9(b) presents a computer simulated reproduction of the kink experimentally observed, providing atomistic and energetic information about the bending process. The overall shape of the kink, along with the distance of the tip of the kink from the upper wall of the tube, is in quantitative agreement with the TEM picture of Figure 9(a). The coding denotes the local strain energy at the various atoms, measured relative to a relaxed atom in an infinite graphene sheet. In all simulations, the same generic features appear: Prior to buckling (≤$30^{\circ }$), the strain energy increases quadratically with the bending angle, corresponding to harmonic deformation [see Figure 9(c)]. In this harmonic regime, the hexagonal rings on the tube surface are only slightly strained and the hexagonal carbon network is maintained. Beyond the critical curvature, the excess strain on the compressed side reaches a maximum and is released through the formation of a kink, which increases the surface area of the bending side. This is accompanied by a dip in the energy *vs.* bending angle curve, as shown in Figure 9(c). Following the kink formation, the strain energy increases approximately linearly until bond breaking occurs under quite large deformation. A similar characteristic energy-strain curve, an initial quadratic curve followed by a linear increase, arises in the case of axial compression [18], as we learned in Section 4.2.

### 6.2. Diameter Dependence

**Figure 10:**
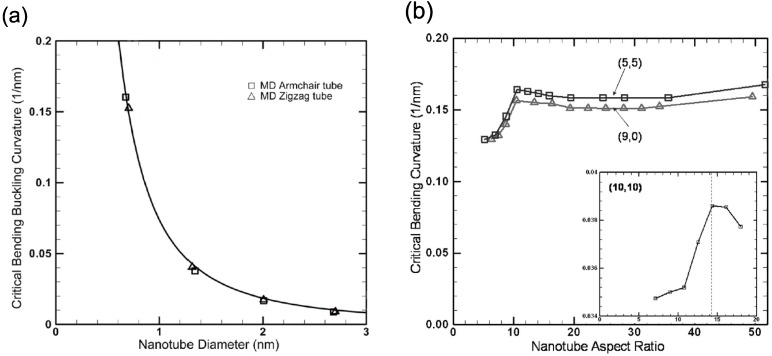
(**a**) Relationship between critical bending buckling curvature $\kappa_{C}$ and nanotube diameter *d* obtained from MD analyses. The tube length is fixed at 24 nm; (**b**) The length/diameter (L/d) aspect ratio dependence of $\kappa_{C}$ for the tube chiralities of (5,5), (9,0), and (10,10). Reprinted from Reference [167].

The geometrical size is a crucial factor for determining buckling behaviors of SWNTs under bending. For instance, those with a small diameter can sustain a large bending angle prior to buckling, and vice versa [167]. Figure 10(a) shows MD simulation data, which show a monotonic increase in the critical curvature $\kappa_{C}$ for with a reduction in the nanotube diameter *d*. The relationship between $\kappa_{C}$ and *d* can be fitted as [18,167] $\kappa_{C}\propto d^{-2}$, which holds regardless of the nanotube chirality.

In addition to the diameter dependence, the critical curvature of SWNTs is affected by the length/diameter (*L*/*d*) aspect ratio. It follows from Figure 10(b) that [167] $\kappa_{C}$ is almost constant for sufficiently long nanotubes such that 10<L/d<50 or more, whereas it drops off for short nanotubes satisfying L/d<10. The critical aspect ratio that separate the two regions is sensitive to the tube diameter [as implied by the inset of Figure 10(b)], but it is almost independent of chirality.

### 6.3. Transient Bending

**Figure 11:**
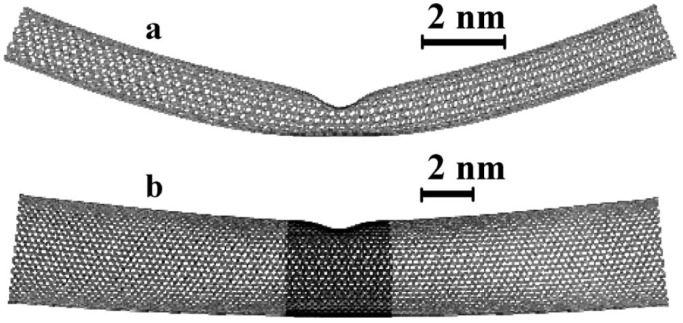
Predicted shape of SWNTs just after buckling, based on MD simulations for (**a**) a 15.7-nm-long (10, 10) SWNT at the bending angle $\theta =43^{\circ }$; and (**b**) a 23.6-nm-long (30, 30) SWNT at $\theta =23^{\circ }$. Note the difference in scale. Reprinted from Reference [68].

We have learned in Section 4 that for relatively thin SWNTs, the buckling is characterized by a discontinuity in the energy curve [Figure 9(c)]. Across the buckling point, the bending angle dependence of the deformation energy changes suddenly from being quadratic in the prebuckling regime to linear in the postbuckling regime. This is, however, not the case for larger diameter nanotubes. As the diameter is increased, a second discontinuity appears in the strain-energy curve at a larger bending angle than the first one [68]. The origin of the two discontinuities can be accounted for by inspection of Figure 11. The bottom image [Figure 11(b)] shows a just-buckled wall of a (30, 30) SWNT corresponding to the first discontinuity in the energy curve, in which the buckled side is far from the opposite side. Therefore, more bending is required to bring the two sides close enough, as observed in Figure 11(a), which results in the second discontinuity.

**Figure 12:**
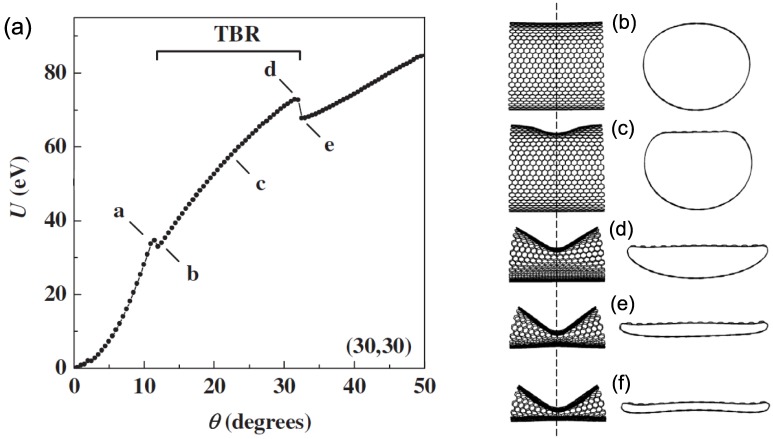
(**a**) Deformation energy *U* for a 23.6-nm-long (30,30) SWNT as a function of the bending angle *θ*. The symbols a–e attached to the curve indicate the points for which the tube shape and cross section at the buckling point are shown in the images (**b**)–(**f**) on the right. “TBR" denotes the transient bending regime. Reprinted from Reference [68].

The thick-nanotube’s buckling behavior mentioned above is illustrated in Figure 12(a), where the deformation energy *U* is plotted as a function of bending angle *θ* for a (30, 30) SWNT [68]. Three distinct deformation regimes are observed, clearly separated by two discontinuities at $\theta =12^{\circ }$ and $32^{\circ }$. In the initial elastic regime, *U* exhibits a quadratic dependence on *θ*, whereas the cross section experiences progressive ovalization as the bending angle increases, culminating to the shape in Figure 12(b). The buckling event is marked by an abrupt transition from the oval cross section to one with the flat top shown in Figure 12(c). As the bending angle increases beyond the first discontinuity [*i.e.*, during the transient bending regime (TBR) indicated in Figure 12(a)], the flat portion of the top wall expands continuously across the nanotube [Figure 12(d,e)] [168]. As a result, the top-to-bottom wall distance decreases gradually, reducing the tube cross section at the buckling site. The deformation energy curve in the TBR is no longer quadratic; in fact, the exponent becomes less than unity. When the approaching opposite walls reach the vdW equilibrium distance of 0.34 nm [Figure 12(f)], the cross section collapses, forming the kink, and a second discontinuity is observed.

## 7. Bend Buckling of MWNTs

### 7.1. Emergence of Ripples

Following the discussions on SWNTs in Section 6, we look into the bend buckling of MWNTs. The difference in the mechanical responses between SWNTs and MWNTs lies in the presence of vdW interactions between the constituent carbon layers. Apparently, thicker MWNTs with tens of concentric walls seem stiffer than few-walled, thin MWNTs against bending, since the inner tubes of MWNTs may reinforce the outer tubes via the vdW interaction. However, the contrary occurs. In fact, whereas MWNTs with small diameter exhibited a bending stiffness of around 1 TPa, those with larger diameter were much more compliant, with a stiffness of around 0.1 TPa [66]. This dramatic reduction in the bending stiffness was attributed to the so-called rippling effect, *i.e.*, the emergence of a wavelike distortion on the inner arc of the bent nanotube [102,103,169,170,171,172,173,174,175,176,177,178,179].

**Figure 13:**
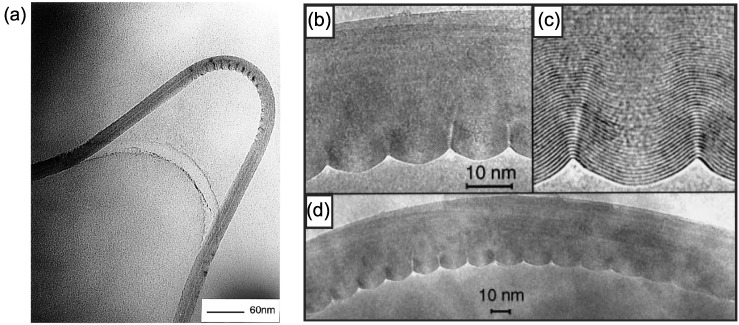
(**a**) Under high bending, MWNTs form kinks on the internal (compression) side of the bend; (**b**)–(**d**) High-resolution TEM image of a bent nanotube (with a radius of curvature of ∼400 nm), showing the characteristic wavelike distortion. The amplitude of the ripples increases continuously from the center of the tube to the outer layers of the inner arc of the bend. Reprinted from References [66,102].

Figure 13(a) presents a clear example of the rippled MWNT structure [102]. The tube diameter is ∼31 nm and it is subjected to a radius of bending curvature of ∼400 nm. Enhanced images at the ripple region [66] are also displayed in Figure 13(b–d), though they are not identical to the specimen in Figure 13(a). The amplitude of the ripple increased gradually from inner to outer walls, being essentially zero for the innermost core tube to about 2 to 3 nm for the outermost wall. Such rippling deformation induces a significant reduction in the bending modulus, as has been explained theoretically by solving nonlinear differential equations [180].

### 7.2. Yoshimura Pattern

**Figure 14:**
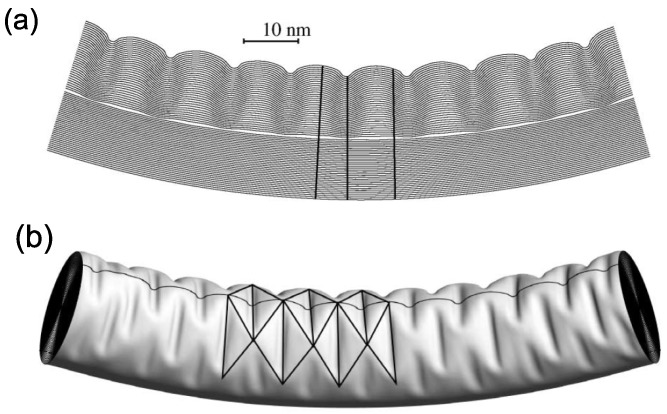
Rippling of a 34-walled carbon nanotube: (**a**) longitudinal section of the central part of the simulated nanotube; and (**b**) the morphology of the rippled MWNT reminiscent of the Yoshimura pattern. Highlighted are the ridges and furrows, as well as the trace of the longitudinal section. Reprinted from Reference [174].

Precise information about the membrane profile and the energetics of the rippling deformation, which are unavailable in experiments, can be extracted from large-scale computer simulations. Figure 14(a) shows a longitudinal cross section of the equilibrium configuration [174]. This image is the computational analog of the TEM slices of rippled, thick nanotubes reported in the literature [66,173]. The simulations reproduce very well the general features of the observed rippled nanotubes: nearly periodic wavelike distortions, whose amplitudes vanish for the inner tubes and smoothly increase toward the outer layer.

A remarkable finding in the simulations is that the rippling deformation closely resembles the Yoshimura pattern [181,182] (a diamond buckling pattern). We can see that the rippling profile in Figure 14(b) consists not of a simple linear sequence of kinks but of a diamond-like configuration of kinks on the compressed side. Such a Yoshimura pattern is well known as characterizing the postbuckling behavior of cylindrical elastic shells on a conventional macroscopic scale. The pattern has the interesting geometric property of being a nearly isometric mapping of the undeformed surface, at the expense of creating sharp ridges and furrows.

The rippling deformation, peculiar to thick MWNTs, is a consequence of the interplay between the strain-energy relaxation and the vdW energy increment. As intuitively understood, the low bending rigidity of individual graphitic sheets, relative to their large in-plane stiffness, makes it possible to release effectively a significant amount of the membrane strain energy at the expense of slight flexural energy. As a result, rippled MWNTs have a significantly lower strain energy than uniformly bent MWNTs.

**Figure 15:**
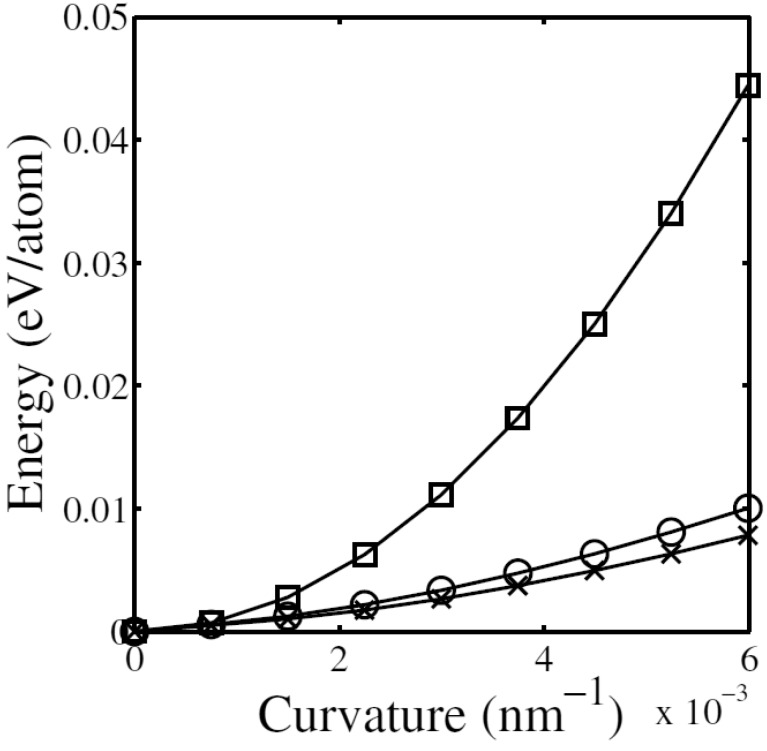
Energy curves for a bent 34-walled nanotube with respect to the bending curvature. Shown are the strain energy for fictitiously uniform bending (squares), the strain energy for actually rippled deformation (crosses), and the total energy (*i.e.*, sum of the strain energy and the vdW one) for rippled deformation (circles). Reprinted from Reference [174].

Figure 15 shows the energy of bent MWNTs as a function of the bending curvature for a 34-walled nanotube [174]. When the nanotube is uniformly bent, the strain energy grows quadratically with respect to the curvature. For such a uniform bending, the vdW energy gives almost no contribution to deformation, and therefore, the total energy also follows a quadratic law. However, the actual behavior of the system greatly deviates from this ideal linearly elastic response. As can be observed in Figure 15, the rippling deformation leads to much lower values of strain energy and an increase in vdW energy. The evolution of the total energy $E_{\mathrm{tot}}$ with respect to curvature radius *R* is very accurately fitted by $E_{\mathrm{tot}}\propto R^{-a}$ with a=1.66; this response differs from that predicted by atomistic simulations of SWNTs or small, hollow MWNTs, both of which exhibit an initial quadratic growth (a=2) in the elastic regime, followed by a linear growth (a=1) in the postbuckling regime. The results in Figure 14 and Figure 15 evidence the failure of the linear elasticity and linearized stability analysis to explain the observed well-defined postbuckling behavior (1<a<2) for thick nanotubes, implying the need for a new theoretical framework based on nonlinear mechanics [183,184,185].

## 8. Twist Buckling

### 8.1. Asymmetric Response of SWNTs

Similarly to bending situations, SWNTs under torsion exhibit a sudden morphological change at a critical torque, transforming into a straight-axis helical shape. The crucial difference from bending cases is that, under torsion, the critical buckling torque of SWNTs depends on the loading direction, *i.e.*, whether the tube is twisted in a right-handed or left-handed manner [186,187]. This load-direction dependence originates from the tube chirality, which breaks the rotational symmetry about the tube axis. For example, the twisting failure strain of chiral SWNTs in one rotational direction may even be 25% lower than that in the opposite direction [187]. Moreover, symmetry breaking causes coupling between axial tension and torsion, giving rise to an axial-strain-induced torsion of chiral SWNTs [188]. This intriguing coupling effect shows that a chiral SWNT can convert motion between rotation and translation, thus promising a potential utility of chiral SWNTs as electromechanical device components.

**Figure 16:**
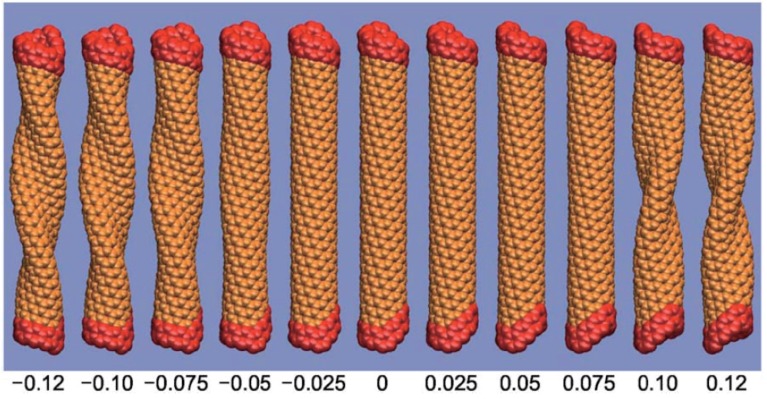
Morphological changes for a (8,3) nanotube under torsion. Applied strain and its direction are indicated beneath the diagram; the digit 0.05, for example, corresponds to the strain of 5%, and the sign + (−) indicates right(left)-handed rotation. Under right-handed rotation, the tube buckles at a critical buckling strain $\gamma_{\mathrm{cr}}$ = 7.6%, whereas it buckles at $\gamma_{\mathrm{cl}}\;=\;4.3\%$ under left-handed rotation. Reprinted from Reference [186].

**Figure 17:**
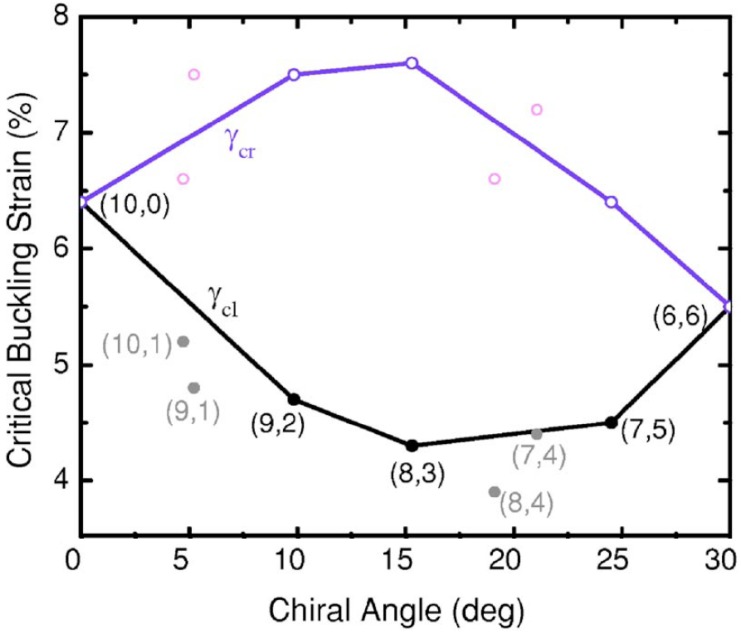
Critical buckling shear strains as a function of tube chirality. Some additional data for SWNTs with slightly larger or smaller diameters are also presented for reference. Reprinted from Reference [186].

The effect of structural details on buckling of a torsional SWNT was explored using MD calculations [186]. Figure 16 shows morphology changes of an (8,3) SWNT under torsion. Its torsional deformation depends significantly on the loading direction. Under right-handed rotation, the tube buckles at a critical buckling strain of ∼7.6%, which is significantly larger than that (∼4.3%) under left-handed rotation. Figure 17 summarizes a systematic computation [186] of the critical buckling strains in both twisting ($\gamma_{\mathrm{cr}}$) and untwisting ($\gamma_{\mathrm{cl}}$) directions as a function of tube chiral angle; a loading-direction-dependent torsional response of chiral tubes is clearly observed. Special attention should be paid to the fact that the maximum difference between $\gamma_{\mathrm{cr}}$ and $\gamma_{\mathrm{cl}}$ is up to 85%. This clear difference in the mechanical response suggests particular caution in the use of carbon nanotubes as torsional components (e.g., oscillators and springs) of nanomechanical devices [117,120,189,190].

### 8.2. Nontrivial Response of MWNTs

**Figure 18:**
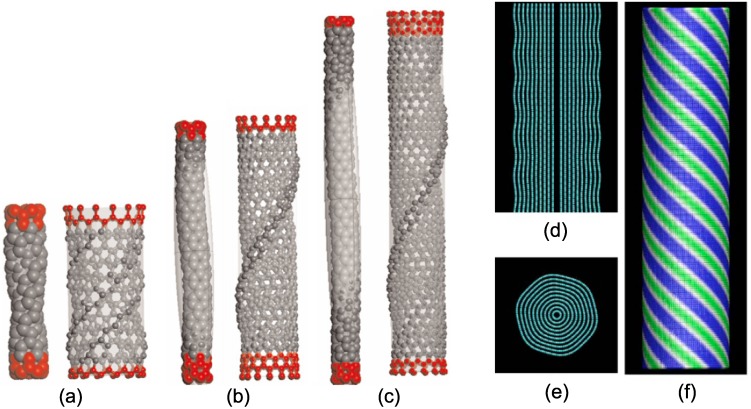
(**a**)–(**c**) Helical buckling of (5,0)@(14,0) DWNTs with lengths: (**a**) L= 1.095 nm; (**b**) 4.45 nm; and (**c**) 6.97 nm. For each tube length, the inner wall shows ordinary buckled patterns, whereas the outer wall exhibits nontrivial buckling modes associated with local rims; (**d**)–(**f**) Helical rippling deformation of a 10-walled nanotube (5,5)@⋯@(50,50) of 34 nm in length and 3.4 nm in radius: (**d**) Longitudinal view; (**e**) Cross-sectional view; (**f**) Deformation map, with green for ridges and blue for furrows. Reprinted from References [75,178].

In contrast to SWNTs cases, fewer studies on MWNTs under torsion were reported because of their complex structures and computational costs. For twisted DWNTs, MD simulations have revealed [75,77] a nontrivial buckling mode involving a few thin, local rims on the outer tube while the inner tube shows a helically aligned buckling mode [Figure 18(a–c)]. These distinct buckling modes of the two concentric tubes imply that a conventional continuum approximation in which it is postulated that the buckling modes of all the constituent tubes have the same shape fails for analyzing the torsional responses of DWNTs.

When increasing the number of constituent walls to far more than two, we acquire torsional rippling deformations [175,177,191,192]. It was numerically found that [178] the amplitude in the torsional rippling of MWNTs can be accurately described by a simple sinusoidal shape function, as confirmed by Figure 18(d,f). It is noteworthy that the characteristics of the helical rippling morphology in twisted MWNTs are different from those in bent MWNTs, *i.e.*, the so-called Yoshimura or diamond buckling pattern. Structurally, torsion-induced rippling is distributed more periodically and uniformly along the tube whereas bending-induced rippling is located only in the compressive side [174,176]. Energetically, in twisted MWNTs, high strain energy is stored along the ridge regions, whereas in bent MWNTs, the strain energy is equally concentrated both at the ridges and furrows.

## 9. Universal Scaling Laws Under Bending and Torsion

**Figure 19:**
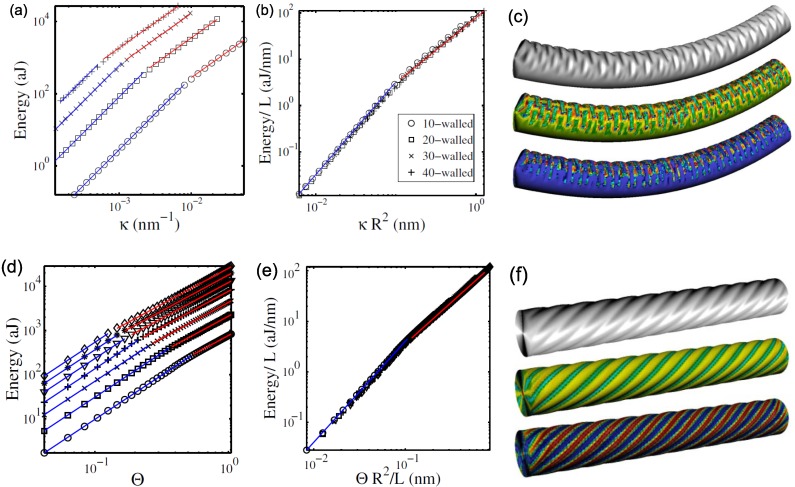
(**a,d**) Strain energy curves as a function of the bending curvature *κ* and the twisting angle Θ; (**b,e**) Data collapse upon appropriate rescaling. The power-law fits with exponents 2 (blue) and a(<2) (red) are shown for illustration. In all four plots, the number of walls increases stepwise from 10 (circles) to 40 (crosses); (**c**) 40-walled nanotube in pure bending; (**f**) 35-walled nanotube in torsion. The latter two panels present the deformed shape (top), Gaussian curvature map (middle, with green being zero, red being positive, and blue being negative), and energy density map (bottom, with red being high and blue being low). Reprinted from Reference [176].

Interestingly, buckled MWNTs under bending and torsion were found to obey universal scaling laws that consist of two distinct power-law regimes in the energy-deflection relation [176]. Figure 19 shows the mechanical response of MWNTs under bending and torsion; the strain energy *E* *vs.* bending curvature *κ* or twisting angle Θ is plotted with the increasing stepwise number of walls from 10 to 40. All the tested MWNTs exhibit two distinct power-law regimes: a harmonic deformation regime characterized by the exponent a=2 for the relation $E\propto \kappa^{a}$ or $E\propto \Theta^{a}$ (indicated by blue lines in Figure 19) and an anharmonic, postbuckling regime with exponent a∼1.4 for bending and a∼1.6 for torsion (red lines). The latter nonlinear response corresponds to the ripples of the graphene walls discussed earlier.

Figure 19(b,e) show the data collapse for all the tested nanotubes upon a universal scaling law. This law is described by the anharmonic exponent *a* and the characteristic length scale $\ell_{\mathrm{cr}}$. In twist situations, it is defined by $\ell_{\mathrm{cr}}=\Theta_{\mathrm{cr}}R^{2}/L$, where *L* and *R* are the length and outer radius, respectively of the MWNTs considered, and $\Theta_{\mathrm{cr}}$ denotes the critical twisting angle at which the buckling arises. In bending cases, $\ell_{\mathrm{cr}}=\kappa_{\mathrm{cr}}R^{2}$ with $\kappa_{\mathrm{cr}}$ being the critical buckling curvature. Then, the unified law plotted in red and blue in Figure 19(b,e) is

(1) $$\frac{E(x)}{L}=\propto \left\{\begin{array}{cc} {\left(xR\right)}^{2} & for\;\;|xR|\leq \ell_{\mathrm{cr}},\\ \ell_{\mathrm{cr}}^{2-a}{\left|xR\right|}^{a} & for\;\;|xR|>\ell_{\mathrm{cr}}, \end{array}\right.$$

with x=ΘR/L or x=κR. The actual value of $\ell_{\mathrm{cr}}$ was evaluated as $\ell_{\mathrm{cr}}∼0.1$ nm for both bending and twisting cases. It should be emphasized that since $\ell_{\mathrm{cr}}$ has dimension of length, the unified law is size dependent; for instance, the thicker the MWNTs, the smaller will be the obtained $\Theta_{\mathrm{cr}}$ or $\kappa_{\mathrm{cr}}$.

## 10. Challenge and Future Directions

In this article, I have provided a bird’s-eye view on the current state of knowledge on the buckling properties of carbon nanotubes. The understanding remains far from complete, but new experiments and theoretical work will no doubt give us a more complete picture and exciting times for both basic and applied research in the realm of nanoscale. Described below are only a few examples of challenging subjects that may trigger innovation in the nanotube research community.

### 10.1. Buckling Effects on Heat Transport

Carbon nanotubes demonstrate the excellent thermal conductivity among any known material. When a carbon nanotube is buckled, however, the localized structural deformation can prohibit ballistic heat transport along the nanotube axis [111]. This results in the decreasing behavior of thermal conductivity of nanotubes under compressive stress, which is attributed to the increase in the phonon-phonon scattering rate. Such the buckling-induced reduction in thermal conductivity has important implications of heat management of nano-scale electronic devices, including the dynamic control of thermal transport and energy conversion. In view of materials sciences, it is also important to make clear the effect of buckling on the thermal properties of carbon nanotube composites [193]. Despite this great potential, very limited number of studies has been conducted on the issue thus far.

From an academic standpoint, the buckling-induced change in the thermal transport poses questions on the feasibility of conventional heat conduction theory for macroscopic solids. It has been clarified that the low-dimensional nature of carbon nanotubes gives rise to various intriguing phenomena: the well-known examples are the lattice soliton based energy transfer [194] and robust heat transport in deformed nanotubes [195]. These phenomena are beyond the classical way of understanding; therefore, it is interesting to consider how each class of nanotube buckling (compressive, bending, torsional, *etc*.) affects the nontrivial heat transport observed in nanotubes. The results obtained will shed light on unexplored problems of thermal conduction in carbon nanotubes and related materials.

### 10.2. Role of Defects and Imperfections

Carbon nanotubes obtained by practical synthesis possess various kinds of defects [11] such as missing atoms (called “vacancy defects"), carbon rings other than usual hexagonal ones (“Stone-Wales defects"), or sp${}^{3}$ bonds instead of usual sp${}^{2}$ bonds (“re-hybridization defects"). Hence, understanding the effect of defects on the mechanical properties of nanotubes is essential in the design of nanotube-based applications. It has been found that these defects can affect considerably the mechanical strength and post-elongated morphology of carbon nanotubes. Comprehensive studies on the influence of defects on their buckling behavior remained, however, lacking in the literature until recently [14].

MD simulations performed in the past few years [196,197,198,199] suggested that the presence of defects, particularly one- or two-atom vacancies, may cause a significant reduction in the buckling capacity. An interesting observation is that the degree of reduction is strongly dependent on the chirality and temperature. For example, in torsional buckling at low temperature, armchair nanotubes are less sensitive to the presence of defects when compared with their zigzag counterparts [198]. Still theoretical investigations have been limited to MD simulations; more accurate and quantitative research based on density-functional methods, for instance, would be desired. Experiments on the defect-induced variance in the buckling behavior are of course to be addressed in future.

Another interesting subject is to employ the presence of defects as regulator of heat conduction through carbon nanotubes. With the increase of number of defects, the thermal conductance of nanotubes rapidly decreases. The reason for this large reduction is that high-frequency phonons which contribute to thermal transport is strongly scattered by the structural defects. It has been numerically predicted that [200] even a few structural defects in nanotubes can lead to a strong suppression of thermal transport by one order of magnitude. This result implies that the structural defects can offer an effective method of tuning thermal transport of carbon nanotubes; such the tuning of heat transport is advantageous in the sense that lattice defects can be well controlled during the growth or by irradiations.

### 10.3. Relevance to Chemical Reaction

Applications of carbon nanotubes in composite materials often require an understanding and control of the chemistry and chemical reactivity of the nanotubes’ sidewall. This is because the carbon-carbon bonding state on the outermost graphitic surfaces determines where the chemically sensitive reactions take place and how the reactions affect the physical properties of carbon nanotubes. For instance, the functionalization and/or chemisorption on the sidewall of nanotubes enable to increase linking between nanotubes as filler and a surrounding matrix. Besides, reactivity control of the nanotube sidewall leads to a novel technique for chemical sensors and drug delivery systems.

An important finding in the context of buckling is that the chemical reactivity of carbon nanotube is dependent on local surface curvature of the outermost sidewall. It has been theoretically demonstrated that [201,202] the reactivity of a nanotube is governed by the local atomic structure of carbon atoms on which the chemisorbing species or functional group can react and/or a stable bond. This results imply the control of sidewall reactivity by artificial deformation; that is, local chemical reactivity of carbon nanotubes can be promoted locally by inducing mechanical deformation like buckling.

It should be reminded that the basic concept of the curvature-dependent reactivity was initially proposed in 1993 [203]; almost twenty years have passed since then. Nevertheless, research progress on the subject seems rather behind [204,205]. Especially, experimental evidence of the proposed reactivity promotion has been scarce, which may be due to difficulty in precise manipulation of nanotube buckling and accurate measurement of adsorption capability on the nanotube sidewall. Considering the importance in view of material science, breakthrough in the chemistry of the deformation-driven reactivity and its possible application is strongly expected.

## Figures and Tables

**Figure A1:**
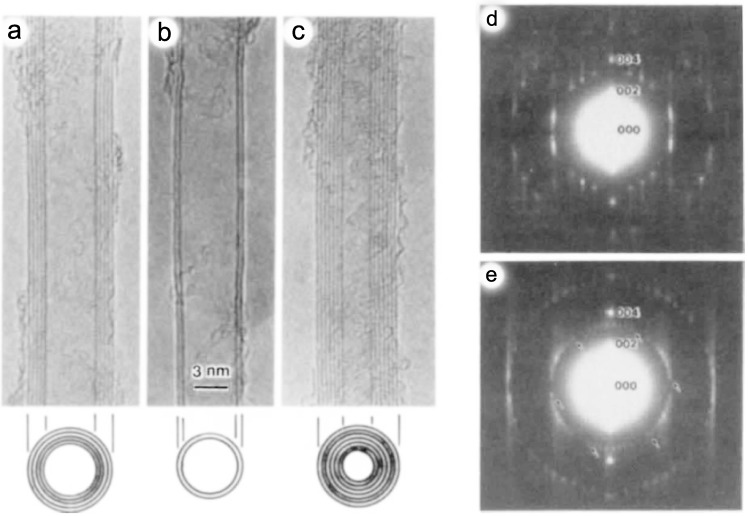
TEM image of nanotubes and sketches of each nanotube’s cross sections for (**a**) a five-wall nanotube with a diameter of 6.7 nm; (**b**) a double-wall nanotube with a diameter of 5.5 nm; and (**c**) a seven-wall nanotube with a diameter of 6.5 nm, which has the smallest hollow diameter (∼2.2 nm) among the three specimens; Electron diffraction patterns showing (**d**) the superposition of three sets of {hk0} spots taken from a seven-wall nanotube; and (**e**) the superposition of four sets of {hk0} spots from a nine-wall nanotube. Reprinted from Reference [3].

**Figure A2:**
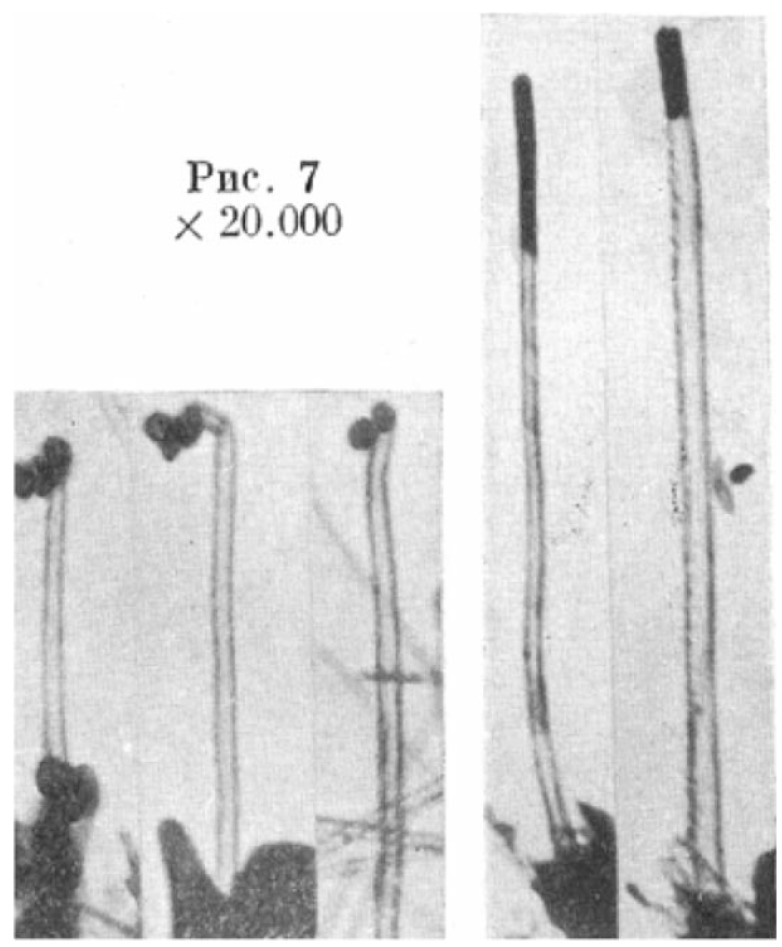
The earliest TEM images of carbon nanotubes published in Reference [209].

## Appendix. Who Discovered Carbon Nanotubes First?

In Appendix, we take a look at the history of who discovered nanotubes and when [206,207,208], for mainly pedagogical reasons. The answer seems obvious at a glance, but it is actually quite profound. The author believes that the argument should be of help for younger readers who may be learning the facts for the first time.

## A1. Iijima’s Nanotube in 1991

As is well known, most academic and popular literature attributes the discovery of carbon nanotubes to Sumio Iijima of NEC in 1991. Without doubt, it is Iijima’s seminal article in 1991 [3], entitled “Helical microtubules of graphitic carbon", that triggered the explosion of carbon nanotube research still fascinating us today. Iijima studied the arc evaporation process that efficiently produced fullerene (C${}_{60}$) molecules. When analyzing a by-product in the arc evaporation, Iijima found large amounts of MWNTs mixed with faceted graphitic particles; see Figure A1 for the image obtained.

From a historical perspective, however, Iijima’s finding is not the first reported carbon nanotube. Careful analysis of the literature shows that there had been many precedents prior to 1991, in which the presence of analogous (and almost identical) nanostructures including “carbon tubes" [209] and “hollow carbon fibers" [210] was uncovered.

## A2. Carbon Nanotubes Prior to 1991

The first evidence for nanosized carbon tubes is believed to have been published in 1952 in a Soviet academic journal [209], almost 40 years before Iijima’s paper. The article, written by Radushkevich and Lukyanovich, demonstrated clear TEM images of tubes (diameter ∼ 50 nm) made of carbon. Figure A2 is a reprint of the image given in Reference [209], which clearly shows carbon filaments exhibiting a continuous inner cavity [211]. However, this discovery was largely unnoticed as the article was published in the Russian language.

Subsequently, many other reports followed this Soviet work. For instance, Oberlin, Endo, and Koyama fabricated in 1976 hollow carbon fibers with nanometer-scale diameters using a vapor-growth technique [210]. In 1979, Abrahamson presented evidence of carbon nanotubes at a conference paper together with their characterization and hypotheses for a growth mechanism [212]. In 1981, a group of Soviet scientists produced carbon tubular crystals and identified them with graphene layers rolled up into cylinders. Moreover, they speculated that by rolling graphene layers into a cylinder, many different arrangements of graphene hexagonal nets, such as an armchair-type and a chiral one, are possible.

## A3. Closing Remarks

In summary, the credit for discovering carbon nanotubes should go to Radushkevich and Lukyanovich. It must be emphasized, however, that merely looking at an object with a microscope is completely different from identifying its structure. That is, Iijima not only observed the nanotubes with an electron microscope but also accurately elucidated its structure from electron diffraction images, and his work was thus on a different level to preceding studies.

Let us return to Figure A1(a–c). The photographs shown here are the first electron micrographs of carbon nanotubes captured by Iijima. Surprisingly, a multiwalled structure with an interlayer spacing of less than 1 nm is clearly observed in this photograph. Even more surprisingly, he measured the electron diffraction shape [Figure A1(d,e)] from a single carbon nanotube. The diffraction strength from a crystal of a light element (carbon) with a thickness of only several nanometers is extremely low, and so, this experiment is not easy to perform even nowadays using the best electron microscopes available. Iijima must have had first-class microscope operating skills to make these measurements in 1991, although he did it with ease. It is also astounding that Iijima identified this substance, which only appeared as a thin, needle-like crystal, as a “tube-shaped substance with a helical structure" based on its electron diffraction shape. How many people in the world would have been able to conclude that it is a nanotube with a helical structure by looking at the electron diffraction shape? Now that we already know the structure of carbon nanotubes, it is not so difficult to read the electron diffraction shape. However, at that time, much experience and sharp insight must have been required by the person observing this electron diffraction shape for the first time in the world to interpret it correctly as a tube-shaped substance with a helical structure.

So, who actually discovered carbon nanotubes? To answer this question, first we need to define the meaning of “discovery of nanotubes". It is probably correct to state that Radushkevich and Lukyanovich were the first to take photographs of the overall picture of the cylindrical tube-shaped nanocarbon substance (although photographs taken by other researchers could still be discovered in future). However, Iijima was the first to reveal that the microscopic structure unique to nanotubes was multiwalled and helical, by using “the eyes" of electron diffraction. The rest is up to the philosophy and preference of the person who is judging. Whatever the judgment, we should never forget that the knowledge and benefits that we now enjoy in nanotube research are the result of the hard work and achievements of our predecessors.
